# TAS-102 in combination with bevacizumab for second-line treatment of metastatic colorectal cancer with a hypertensive elderly patient: a case report

**DOI:** 10.3389/fonc.2025.1558470

**Published:** 2025-03-28

**Authors:** Lingling Xu, Xun Qiu, Hongmei He, Lili Liu, Qing He, Jinghua Sun

**Affiliations:** Department of Medical Oncology, Second Affiliated Hospital of Dalian Medical University, Dalian, China

**Keywords:** TAS-102, bevacizumab, case report, metastatic colorectal cancer, mCRC, elderly patient, second-line

## Abstract

**Background:**

Colorectal cancer (CRC) is a common malignant tumor worldwide. Approximately 20%–25% of patients have metastases at the time of initial diagnosis, and nearly half eventually develop metastatic cancer. The standard first- and second-line treatments for unresectable metastatic CRC are full-dose two-/three-agent chemotherapy with or without a combination of molecularly targeted agents. However, many patients are ineligible for intensive therapy due to poor performance status or advanced age. TAS-102 (trifluridine/tipiracil) in combination with bevacizumab may provide a new treatment strategy for patients with advanced CRC who are ineligible for intensive therapy.

**Case report description:**

We report a case of a 91-year-old woman diagnosed with stage IV adenocarcinoma of the rectosigmoid junction in the presence of multiple metastases. The patient had a history of hypertension, had suffered from deep vein thrombosis of the left lower extremity, and was allergic to several drugs. Genetic testing showed multiple mutations in Kirsten Rat Sarcoma Viral Oncogene Homolog (KRAS), Phosphatidylinositol-4,5-bisphosphate 3-Kinase Catalytic Subunit Alpha (PIK3CA), and Tumor Protein p53 (TP53); microsatellite stability; and a tumor mutational burden of 4.5 Mut/Mb. The patient was diagnosed with stage IV adenocarcinoma of the rectosigmoid junction in May 2020, with a clinical stage of cTxNxM1. Without surgery, the patient received first-line treatment with capecitabine in combination with bevacizumab, which was changed to second-line treatment with TAS-102 in combination with bevacizumab after disease progression, with a progression-free survival of 10 months, achieving a significant survival benefit. Later, due to the patient’s poor physical condition, no further medication was administered, and the patient died on 1 September 2022.

**Conclusion:**

TAS-102 in combination with bevacizumab for the treatment of elderly patients with metastatic CRC who are ineligible for intensive therapy is a promising treatment option.

## 1 Introduction

According to the statistics from GLOBOCAN 2022, more than 1.92 million new cases of colorectal cancer (CRC) are diagnosed worldwide each year, with over 900,000 deaths, ranking third in new cancer cases and second in causes of cancer death globally (1). In China, CRC also brings a heavy disease burden, with more than 500,000 new cases per year, ranking second among all new cancer cases, and more than 240,000 deaths, with the number of deceased patients ranking fourth among all cancers (2). Due to the atypical early symptoms of CRC, approximately 20%–25% of patients with CRC have metastasis at the time of initial diagnosis, and nearly half eventually progress to metastatic cancer (3, 4).

For unresectable metastatic colorectal cancer (mCRC), the standard first-line and second-line treatments involve full-dose dual-agent/triple-agent chemotherapy combined with or without molecularly targeted drugs. However, the median age of patients with CRC at diagnosis is 68 years old, with more than 60% of patients over 65 years old at initial diagnosis, and older patients are more susceptible to underlying conditions such as hypertension, diabetes, and cardiovascular and cerebrovascular diseases. Thus, many of them are ineligible for intensive therapy due to poorer performance status (5–7).

Trifluridine/tipiracil (FTD/TPI, under the developmental codename TAS-102) is an oral combination drug of antineoplastic nucleoside analogs based on thymidine, consisting of 1 mole (M) of FTD and 0.5M TPI (8, 9). After phosphorylation, FTD can be incorporated into the deoxyribonucleic acid (DNA) of tumors, leading to DNA dysfunction and thereby inhibiting cell proliferation, and TPI can inhibit the degrading action of thymidine phosphorylase on FTD (9, 10). The results from the international, multicenter, randomized, double-blind, placebo-controlled, phase III RECOURSE study confirm that, compared to placebo, TAS-102 provides a statistically significant survival benefit for patients with mCRC, and the safety profile is consistent with previously reported studies (11). In the treatment of mCRC, the continuous inhibition of angiogenesis may be an effective strategy. Several phase II clinical trials have demonstrated the survival benefits of TAS-102 in combination with bevacizumab (12–14). The international multicenter, open-label, randomized, controlled phase III SUNLIGHT study, which enrolled a total of 492 patients, was designed to compare the efficacy and safety of TAS-102 in combination with bevacizumab versus TAS-102 alone in the third-line treatment of patients with refractory mCRC. The study results showed that compared with the TAS-102 monotherapy group, the median overall survival (OS) [7.5 vs. 10.8 months, hazard ratio (HR)=0.61, 95% confidence interval (CI), 0.49–0.77; P<0.001] and median progression-free survival (PFS) (2.4 vs. 5.5 months, HR=0.44, 95%CI, 0.36–0.54; P < 0.001) of the TAS-102 combined with bevacizumab group were both significantly prolonged. The study result confirms that TAS-102 in combination with bevacizumab is an efficacious treatment option for patients with refractory mCRC and is independent of mutation status, tumor location, and whether the patient has received prior treatment with bevacizumab (15). The efficacy and safety of TAS-102 in combination with bevacizumab was also evaluated in patients with mCRC who were ineligible for intensive therapy. The phase III SOLSTICE study results indicate that in the population of mCRC patients who were ineligible for intensive therapy, the PFS of first-line treatment with TAS-102 combined with bevacizumab does not show superiority over capecitabine combined with bevacizumab. However, the risk of death in both treatment groups was similar, and TAS-102 combined with bevacizumab had a manageable safety profile distinct from that of capecitabine combined with bevacizumab, making it a viable alternative for those who are ineligible for intensive therapy (16, 17). Based on the results of the phase III SOLSTICE clinical study, the CSCO Colorectal Cancer Diagnosis and Treatment Guidelines (2024) recommend TAS-102 in combination with bevacizumab as a first-line treatment for patients with mCRC who are ineligible for intensive therapy (level III recommendation) (18).

Herein, a case is reported of an elderly patient with mCRC who achieved a significant survival benefit after 10 months of second-line treatment with TAS-102 in combination with bevacizumab following disease progression on first-line therapy.

## 2 Case report

### 2.1 Initial diagnosis data

**Figure 1:**
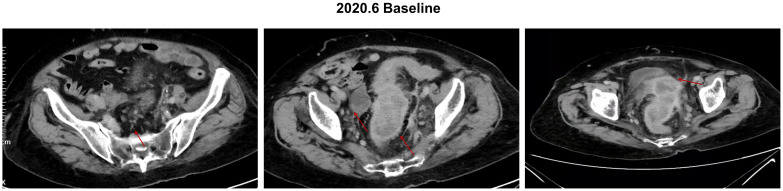
Baseline abdominal CT image of the patient, with red arrows representing the location of the lesions. Baseline conditions: lymph nodes 12*8mm, rectum lesion 42*72mm, right adnexa lesion 36*21mm, and pelvic peritoneum lesion 38*28 mm.

The patient was a 91-year-old woman (height of 162 cm and weight of 65 kg) with an Eastern Cooperative Oncology Group Performance Status (ECOG PS) score of 1 who suffered from hypertension, had previous left lower extremity deep vein thrombosis, had undergone interventional surgery for liver cysts, and was allergic to penicillin and cephalosporins, with no family history of disease. The patient presented with diarrhea symptoms in early 2020, with occasional blood in the stool, which was not taken seriously until she went to the hospital after frequent diarrhea symptoms. In May 2020, colonoscopy results showed an infiltrating cauliflower-like mass growth 15 cm from the anus, invading three-quarters of the circumference of the colon, which was diagnosed as a progressive stage of colorectal cancer by endoscopy. Abnormalities of the sigmoid colon and part of the rectum were diagnosed by abdominal computed tomography (CT), which was consistent with the manifestation of colorectal cancer, accompanied by invasion of the peripheral lymph nodes, metastasis of the right adnexa, metastasis of the pouch of Douglas, and metastasis of the peritoneum in the pelvic cavity (**Figure 1**). The genetic testing results showed mutations in KRAS, PIK3CA, TP53, Isocitrate Dehydrogenase 2 (IDH2), CREB Binding Protein (CREBBP), Colony Stimulating Factor 1 Receptor (CSF1R), GATA Binding Protein 1 (GATA1), Membrane Associated Guanylate Kinase, WW and PDZ Domain Containing 2 (MAGI2), and Transcription Factor 7 Like 2 (TCF7L2). with microsatellite stability and a tumor mutational burden of 4.5 Mut/Mb. The final diagnosis was adenocarcinoma of the rectosigmoid junction stage IV, cTxNxM1.

### 2.2 First-line treatment

**Figure 2:**
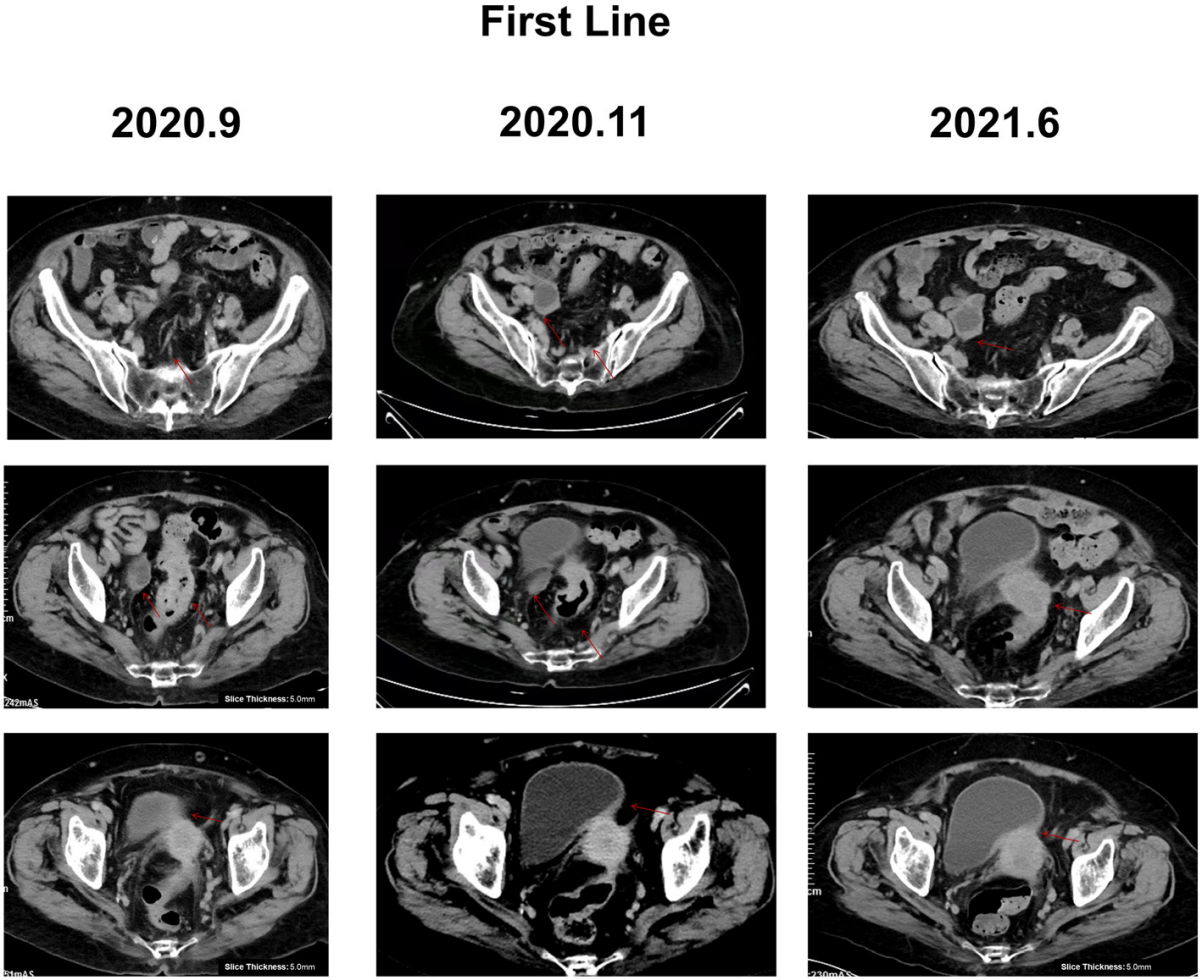
CT image of the abdomen of the patient during first-line treatment, with red arrows representing the location of the lesions. During first-line treatment: lymph nodes reduced to 10*8mm, rectum lesion reduced to 34*32mm, right adnexa lesion 36*21mm, and pelvic peritoneum lesion 10*13 mm. June 2021: disease progression, rectum lesion enlarged to 48*40mm, right adnexa lesion enlarged to 35*30mm, bladder invaded, and pelvic peritoneum lesion 10*13 mm.

Due to advanced age and the family’s desire for conservative treatment, the patient did not undergo surgery and received 19 cycles of capecitabine combined with bevacizumab from June 2020 to June 2021. Additionally, due to the patient’s hypertension and medical history, cardiovascular monitoring was conducted in real time. At this time, the diagnosis was adenocarcinoma of the rectosigmoid junction stage IV, cTxNxM1, and the patient suffered from grade 3 hypertension. After receiving one cycle of capecitabine monotherapy, the patient started combination therapy with bevacizumab in the second cycle, which was tolerated. Following the improvement in disease symptoms, based on the capecitabine prescribing information, the dosage was increased to 1,000 mg in the morning and 1,500 mg in the evening. On the 8th day, the patient experienced rectal bleeding and difficulty in defecation, so capecitabine was not continued for the rest of the cycle. In the third cycle, the dose of capecitabine was reduced to 1,000mg in the morning and 1,000mg in the evening, and the combination therapy with bevacizumab continued. In September 2020, the patient developed mild lower left limb edema and was treated with rivaroxaban for anticoagulation. Inferior vena cava filter placement was recommended at the consultation, which was refused by the family. After the ninth cycle, the patient’s lower limb edema had worsened compared to before. Considering the patient’s advanced age, the presence of deep vein thrombosis in the lower limbs, and concurrent anti-angiogenic therapy, there was a bleeding risk associated with rivaroxaban for anticoagulation. Based on the bevacizumab prescribing information, bevacizumab targeted therapy was discontinued from the 9–10 cycle, and the dose of capecitabine was increased to 1,500mg in the morning and 1,500mg in the evening. After the 10th cycle, the patient’s symptoms improved, so in cycles 11 to 19, the combination of capecitabine and bevacizumab was resumed. In June 2021, a follow-up examination revealed an increase in tumor markers (**Supplementary Figure 1**) and a worsening of tenesmus symptoms compared to before, indicating disease progression (**Figure 2**).

### 2.3 Second-line treatment

**Figure 3:**
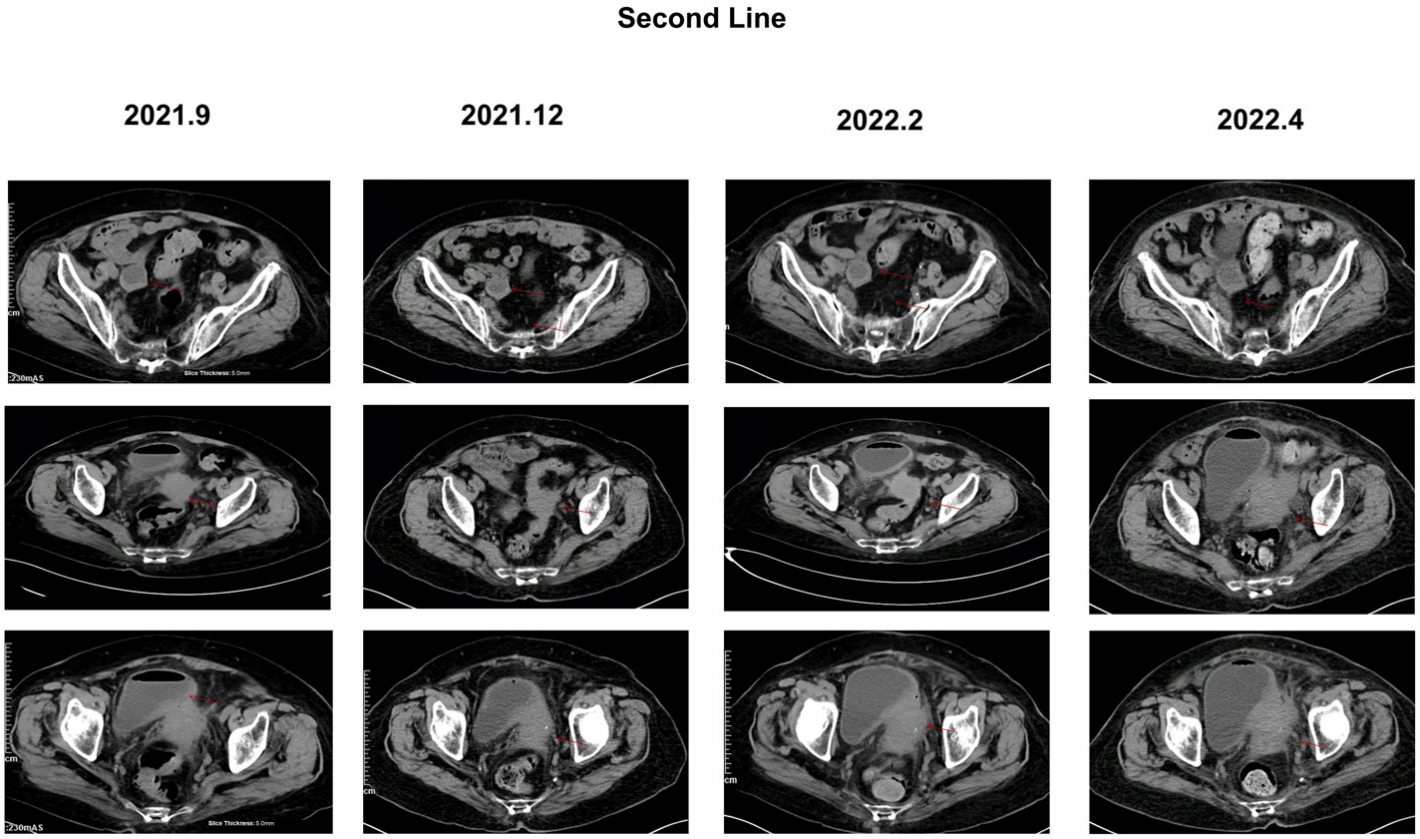
CT image of the abdomen of the patient during second-line treatment, with red arrows representing the location of the lesions. During second-line treatment: rectum lesion reduced to 45*37mm, right adnexa lesion 35*30mm, and pelvic peritoneum lesion 11*13 mm. February 2022: rectum lesion 60*37mm, right adnexa lesion 35*30mm, bladder invasion, and pelvic peritoneum lesion 11*13 mm. April 2022: disease progression, rectum lesion enlarged to 76*52mm, right adnexa lesion 39*30mm, bladder invaded and thickened, and pelvic peritoneum lesion enlarged to 17*13 mm.

**Figure 4:**
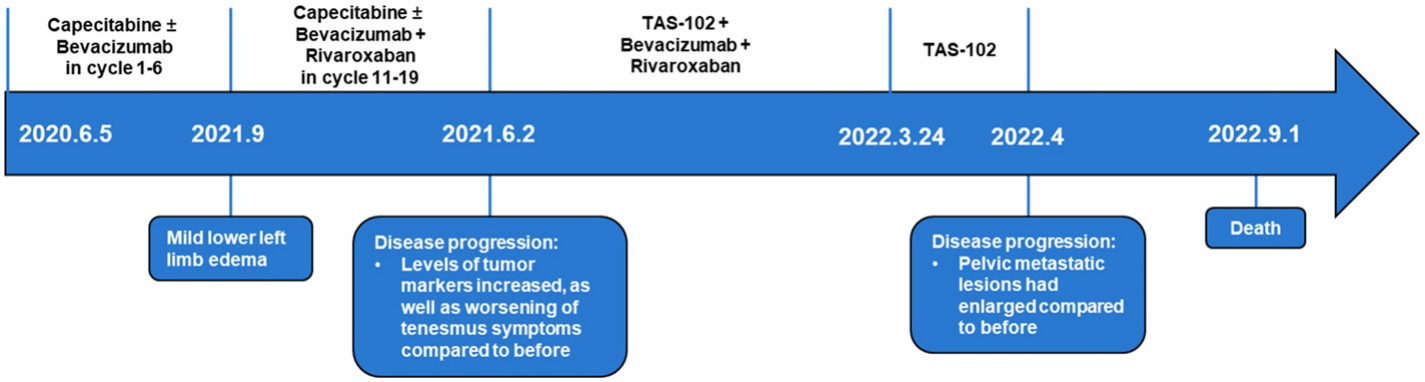
The complete treatment regime and progression of the patient.

From June 2021 to March 2022, the patient received TAS-102 in combination with bevacizumab targeted therapy. At this time, the diagnosis was adenocarcinoma of the rectosigmoid junction stage IV, cTxNxM1, accompanied by grade 3 hypertension and left femoral superficial vein thrombosis. In the follow-up examination in February 2022, it was found that the pelvic metastatic lesions had slightly increased in size and there was an increase in tumor markers (**Figure 3**). In mid-March 2022, the patient experienced hematuria and grade 3+ proteinuria, thus, rivaroxaban tablets and bevacizumab were discontinued. Due to the COVID-19 pandemic, the patient did not return to the hospital and received monotherapy with TAS-102. In April 2022, the symptoms of hematuria improved, but the follow-up examination revealed that the pelvic metastatic lesions had enlarged compared to before, invading the ureter, with left renal pelvis hydronephrosis, indicating disease progression. An examination showed proteinuria 3 + and occult blood +, and a routine blood test indicated a white blood cell count of 3.34 * 10^9^/L, neutrophils of 2.03 * 10^9^/L, and Hb of 69 g/l. Moreover, adverse reactions included moderate anemia, fatigue, poor appetite, loss of physical strength, frequent urination, urgency of urination, and tenesmus, among others. Due to the patient’s poor physical condition, no further medical treatment was administered, and the patient passed away on 1 September 2022. All treatment procedures and outcomes are summarized in **Figure 4**.

## 3 Discussion

This case report presents a 91-year-old patient with KRAS-mutant mCRC who also suffered from hypertension, making her ineligible for intensive treatment. In addition to focusing on the treatment of CRC, real-time monitoring of other health indicators, such as cardiovascular status, was conducted. The patient did not undergo surgery and initially received first-line treatment with a combination of capecitabine and bevacizumab. Due to disease progression, the patient then received second-line treatment with TAS-102 in combination with bevacizumab, achieving a PFS of 10 months, which brought significant survival benefits. During the treatment period, the patient mainly experienced hematological adverse events (AEs), including anemia, leukopenia, and fatigue, with a tolerable safety profile consistent with previous safety profiles that occurred during treatment with TAS-102 in combination with bevacizumab.

In recent years, CRC has become a common malignant tumor. In China, the number of new cases of CRC has remained high for a long time, and it has become one of the tumors that seriously threaten the health of the nation (19). As a complex disease, CRC is driven by a variety of genetic and epigenetic alterations that disrupt critical signaling pathways. Pathways such as EGFR/MAPK, PI3K/Akt, Notch, TGF-β, and Wnt are closely associated with the pathogenesis of CRC, regulating processes including cell proliferation, survival, apoptosis, and metastasis. Dysregulation of these pathways can lead to uncontrolled cell growth and tumor formation (20). CRC symptoms are not obvious in the early stages of the disease, and the disease progresses to a certain level before it is taken seriously. Nearly a quarter of patients have metastases at the time of the initial diagnosis, and nearly half of them eventually develop metastatic cancer (3, 4, 21). The primary treatment for advanced CRC is systemic drug therapy. Two/three-agent chemotherapy FOLFOX/FOLFIRI/FOLFOXIRI in combination with targeted drugs, either anti-VEGF monoclonal antibody or anti-EGFR monoclonal antibody, is the standard first-/second-line treatment option for mCRC (18).

In conventional treatment, only 40% to 50% of patients are eligible for intensive therapy (22, 23). In general, patients who are ineligible for intensive treatment are often those who are older, have poor physical status, and have a higher number of comorbidities. Japanese treatment guidelines state that patients inappropriate for intensive systemic therapy (vulnerable) include those with some comorbidities, those who are considered intolerant to first-line therapy with oxaliplatin and irinotecan, and those who are considered intolerant to concomitant therapy with molecular targeted drugs (24). A previous meta-analysis showed that for patients who are ineligible for intensive chemotherapy due to advanced age or frailty, the median OS associated with such treatment was only 20.4 months (95% CI, 17.3–24.8) (25). Another study has shown that fluorouracil drugs are associated with cardiotoxicity-related diseases, such as angina pectoris, in a certain proportion of patients (26). In this case, the safety risks associated with choosing a standard second-line chemotherapy regimens, such as two-agent chemotherapy in combination with targeted therapy, were high due to the elderly age of the patient, which again demonstrates the need to broaden the range of treatment options for this patient population. An open-label, randomized, phase III AVEX study evaluated the efficacy and safety of capecitabine in combination with bevacizumab for the first-line treatment of elderly patients with mCRC. A total of 280 elderly patients with a median age of 76 were enrolled in the study. The results showed that the median OS for elderly patients with mCRC treated with capecitabine in combination with bevacizumab was 20.7 months (95% CI: 17.0-26.0), the median PFS was 9.1 months (95% CI: 7.3-11.4), and the disease control rate (DCR) was 74% (27). In this case report, the patient initially received first-line treatment with capecitabine combined with bevacizumab, achieving a PFS benefit of nearly 12 months, which is superior compared to the results of the aforementioned study. Another open-label, randomized, phase III SOLSTICE study, which included 856 patients, 426 of whom received TAS-102 in combination with bevacizumab, was designed to evaluate the efficacy of TAS-102 in combination with bevacizumab versus capecitabine in combination with bevacizumab for first-line treatment of patients with unresectable mCRC who were unable to undergo intensive therapy. The results showed that the median PFS in the TAS-102 combined with bevacizumab and capecitabine combined with bevacizumab groups was 9.4 months (95% CI, 9.1–10.9) vs. 9.3 months (95% CI, 8.9–9.8) (HR=0.87; 95% CI, 0.75–1.02), respectively, and the median OS was 19.74 months (95% Cl, 18.04–22.40) vs. 18.59 months (95% Cl, 16.82–21.39) (HR=1.06; 95% CI, 0.90–1.25), respectively. While the PFS of TAS-102 in combination with bevacizumab for mCRC patients unsuitable for intensive therapy was not superior to that of capecitabine in combination with bevacizumab as the risk of death was similar in both groups, the treatment has a manageable safety profile that differs from that of capecitabine in combination with bevacizumab, and, as such, may serve as a viable alternative to it (16, 17). Based on the results of the SOLSTICE study, TAS-102 in combination with bevacizumab has been recommended by the CSCO guidelines for the first-line treatment of mCRC patients who are ineligible for intensive therapy (18). For patients who are ineligible for intensive therapy, there is less research data on second-line and subsequent lines of therapy.

A subgroup analysis of the RECOURSE study showed that the efficacy and safety in patients ≥65 years of age treated with TAS-102 were similar to those reported in those <65 years of age (11). An age-based *post hoc* analysis of the SUNLIGHT trial showed that treatment with TAS-102 in combination with bevacizumab demonstrated benefits in terms of OS, PFS, and delayed deterioration of ECOG PS regardless of the age of patients with refractory mCRC. In the <65, 65–74, and ≥75 years age groups, the most common AEs during TAS-102 combined with bevacizumab treatment included neutropenia (58.2%, 67.1%, and 70.8%, respectively), nausea (41.1%, 30.3%, and 33.3%, respectively), anemia (27.4%, 30.3%, and 33.3%, respectively), malaise (22.6%, 25.0%, and 33.3%, respectively), decreased appetite (15.8%, 25.0%, and 33.3%, respectively), fatigue (21.2%, 23.7%, and 16.7%, respectively), and decreased neutrophil counts (11.6%, 15.8%, and 20.8%, respectively), and the incidence of AEs was similar and well-tolerated among the age groups during treatment (28). The retrospective WJOG14520G study was performed to evaluate the efficacy and safety of patients with mCRC who had previously received treatment and were intolerant to intensive therapy (vulnerable). Out of the 93 patients enrolled in the study, 63% (59 patients) had RAS mutations, and 80% (74 patients) received TAS-102 in combination with bevacizumab as a second-line therapy. The study results showed a median OS of 18.6 months (95% CI, 12.1–23.2), median PFS of 6.3 months (95% CI, 5.0–8.3), and DCR of 67.9% (95% CI, 56.6%–77.8%). This also demonstrates the potential efficacy and acceptable safety profile of TAS-102 in combination with bevacizumab for the treatment of previously treated mCRC patients who are ineligible for intensive therapy (vulnerable) (29). In this case report, the patient achieved a PFS benefit of 10 months after receiving second-line therapy with TAS-102 combined with bevacizumab. This outcome is comparable to the survival benefits reported in other case reports for elderly patients with mCRC and hypertension undergoing second-line treatment, aligning with the clinical expectations of previous treatments (30, 31). Combining evidence-based medicine with clinical practice experience, for elderly patients or those with poor baseline conditions, TAS-102 in combination with bevacizumab can be considered a safe and effective clinical treatment option. Due to the limited clinical trial data available for second-line treatment in this patient population, the CSCO guidelines have not yet provided specific recommendations in this regard. Although this case report demonstrates a considerable survival benefit, data from large-sample prospective studies are still needed for validation.

In this case report, the patient did not receive further subsequent treatments due to poor physical condition in the later stages. For patients who are ineligible for intensive therapies, the selection of third-line and subsequent treatment regimens is particularly crucial. In the WJOG14520G study, of the 89 patients who completed treatment with TAS-102 combined with bevacizumab, 42 patients opted for subsequent therapy. Among them, 16 patients received treatment with the small molecule tyrosine kinase inhibitor (TKI) regorafenib (29). In recent years, the later-line treatment of mCRC has been evolving towards a more personalized and diversified approach. Studies have also observed that the combination of TAS-102 with small-molecule TKI agents shows potential benefits for patients with mCRC. The phase II REGTAS study demonstrated that the combination of TAS-102 and regorafenib in treating patients with mCRC resulted in a median PFS of 4.9 months (95% CI, 2.8-7.0) and a DCR of 84.6% (95% CI, 54.6%-98.1%), indicating promising efficacy (32).

The majority of elderly patients with CRC also have other comorbidities. During treatment, the advanced age of the patients and their comorbidities increase the complexity and risk of treatment, so personalized treatment is particularly important for these patients. In this case, when the patient presented with left lower limb edema and an increased risk of thrombosis, the timely adjustment of medication dosage was used to alleviate symptoms. The selection and adjustment of the treatment plan fully reflect the importance of personalized treatment. In addition, the physical status and adverse reactions of the patients need to be considered, and the treatment program needs to be appropriately adjusted at different stages, combined with consultation with a multidisciplinary team, to further improve the efficacy of the treatment, and while treating, attention should also be paid to nutritional support to enhance the patient’s quality of life and improve adherence.

## 4 Conclusion

A 91-year-old patient with mCRC and hypertension, through a personalized treatment strategy, received second-line treatment with TAS-102 in combination with bevacizumab, achieving a PFS of 10 months, which brought significant survival benefits. This case demonstrated that TAS-102 in combination with bevacizumab could be a promising treatment option for patients with mCRC who are ineligible for intensive therapy. We look forward to future large-sample studies to further validate its efficacy.

## Data Availability

The original contributions presented in the study are included in the article/**Supplementary Material**. Further inquiries can be directed to the corresponding author.
