# Supplementary material for: TAS-102 in combination with bevacizumab for second-line treatment of metastatic colorectal cancer with a hypertensive elderly patient: a case report
Source: Front Oncol. 2025 Mar 28;15:1558470. doi: 10.3389/fonc.2025.1558470 (PMC11985432; doi:10.3389/fonc.2025.1558470)
Supplement: Supplementary file 1 [file Image1.pdf]

Supplementary Material

Supplementary Figure

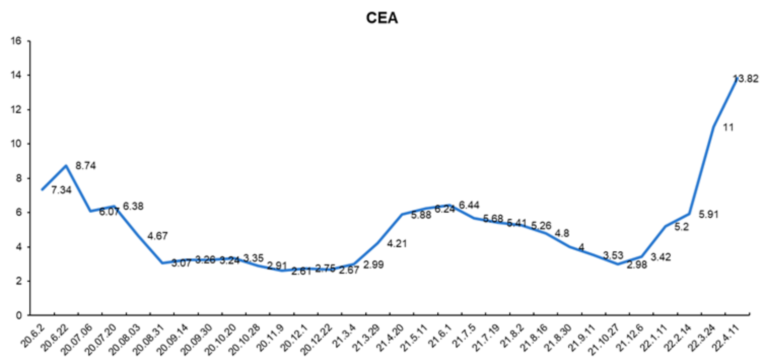

Supplementary Figure 1. Carcinoembryonic antigen (CEA) level during treatment period
